# Circulating Natural Autoantibodies to HER2-Derived Peptides Performed Antitumor Effects on Oral Squamous Cell Carcinoma

**DOI:** 10.3389/fphar.2021.693989

**Published:** 2021-11-05

**Authors:** Xiu Liu, Ziyi He, Yi Qu, Qingyong Meng, Lizheng Qin, Ying Hu

**Affiliations:** ^1^ Beijing Institute of Dental Research, Beijing Stomatological Hospital, Capital Medical University, Beijing, China; ^2^ Department of Transfusion Research, Dongguan Blood Center, Dongguan, China; ^3^ Department of Oral and Maxillofacial and Head and Neck Oncology, Beijing Stomatological Hospital, Capital Medical University, Beijing, China; ^4^ Laboratory for Nursing Science and Institute of Laboratory Medicine, Guangdong Medical University, Dongguan, China

**Keywords:** circulating natural autoantibodies, HER2, oral squamous cell carcinoma, trastuzumab, intravenous immunoglobulin

## Abstract

Natural autoantibodies play a crucial role in destruction of malignant tumors due to immune surveillance function. Epidermal growth factor receptor 2 (HER2) has been found to be highly expressed in a variety of epithelial tumors including oral squamous cell carcinoma (OSCC). The present study was thus undertaken to investigate the effect of anti-HER2 natural autoantibodies on OSCC. Compared with cancer-adjacent tissues, cancer tissues from OSCC patients exhibited higher HER2 expression especially in those with middle & advanced stage OSCC. Plasma anti-HER2 IgG levels examined with an enzyme-linked immunosorbent assay (ELISA) developed in-house showed differences between control subjects, individuals with oral benign tumor and patients with OSCC. In addition, anti-HER2 IgG-abundant plasma was screened from healthy donors to treat OSCC cells and to prepare for anti-HER2 intravenous immunoglobulin (IVIg). Both anti-HER2 IgG-abundant plasma and anti-HER2 IVIg could significantly inhibit proliferation and invasion of OSCC cells by inducing the apoptosis, and also regulate apoptosis-associated factors and epithelial-mesenchymal transition (EMT), respectively. Besides, the complement-dependent cytotoxicity (CDC) pathway was likely to contribute to the anti-HER2 IgG mediated inhibition of OSCC cells. After the HER2 gene was knocked down with HER2-specific siRNAs, the inhibitory effects on OSCC cell proliferation and apoptotic induction faded away. In conclusion, human plasma IgG, or IVIg against HER2 may be a promising agent for anti-OSCC therapy.

## Introduction

Oral squamous cell carcinoma (OSCC) is the sixth leading cancer worldwide and accounts for approximately 90% of all oral malignant tumors (Niu et al., 2020; Zheng et al., 2021). It has been estimated that there are about 400,000 new cases diagnosed as having OSCC with 170,000 OSCC-related deaths each year (Sasahira and Kirita, 2018; Sarkar et al., 2021; Yang et al., 2021). Most people with OSCC already progressed to the advanced stage when they were diagnosed (Sasahira and Kirita, 2018). About 20–30% OSCC patients experienced relapse regardless of treatment; the prognosis of OSCC is rather poor and the overall 5-years survival rate is roughly 50% across the globe (Singhvi et al., 2017; Zhao et al., 2020; Sarkar et al., 2021). Current therapies available for treatments of OSCC include surgical resection, radiotherapy, chemotherapy, immunotherapy, gene therapy as well as combination of several therapies (Gau et al., 2019; Gigliotti et al., 2019; Yang et al., 2020); however, these treatments may result in serious problems such as complications, oral cavity disorders and multi-drug resistance (MDR) to chemotherapy (Chen et al., 2016).

Human epidermal growth factor receptor (EGFR) 2, also called HER2, is a member of the EGFR family of transmembrane receptor tyrosine kinase, and has been reported to be associated with cell growth and survival (Huang et al., 2018; Mirza et al., 2020). HER2 is a non-ligand binding member and exerts its activity by heterodimerization with other EGFR family members, leading to the initiation of multiple signaling pathways involved in cellular proliferation and tumorigenesis (Huang et al., 2018; Oh and Bang, 2020). High HER2 expression due to gene amplification drives oncogenic signaling in various organs and tissues of cancer origin including esophagogastric, breast, head, and neck and other types of cancer (Cierpikowski et al., 2018; Grenda et al., 2020; Pennacchiotti et al., 2021; Sanz-Moreno et al., 2021). Meanwhile, diverse results regarding the overexpression of HER2 in OSCC have been reported (Werkmeister et al., 2000; Fong et al., 2008; Cierpikowski et al., 2018). This highlights the importance of further investigation of HER2 in OSCC development. Currently, small molecule inhibitors or antibodies targeting HER2 have been approved for treatment of patients with HER2-positive breast cancer, non-small cell lung cancer (NSCLC), and gastroesophageal cancer (Iqbal and Iqbal, 2014; Cameron et al., 2017; Kneissl et al., 2017; Liu et al., 2018; Okamoto et al., 2020). Two FDA-approved HER2 monoclonal antibodies (mAbs), namely trastuzumab, and pertuzumab, have shown anti-tumor effects by attacking HER2 signaling (Gerson et al., 2017). Trastuzumab has been used clinically for the treatment of metastatic breast cancer (Kristeleit et al., 2016; Early Breast Cancer Trialists' Collaborative, 2021). Pertuzumab is a recombinant humanized mAb binding to the extracellular dimerization domain II of HER2 to prevent the ligand-induced HER2 heterodimer formation and reduce the survival of tumor (Barthelemy et al., 2014). However, mAb-based immunotherapy has raised new questions about assessment of drug toxicity, the economics of cancer therapy, and resistance to treatments (Kristeleit et al., 2016; Dempsey et al., 2021). Hitherto, HER2-targeted medications have not been applied clinically to treat OSCC, so that there is an urgent need to develop HER2-based alternative and safe therapies.

Natural autoantibodies are defined as immunoglobulins constitutively produced by B-1 cells in the absence of external antigen stimulation (Schwartz-Albiez et al., 2009; Panda and Ding, 2015), and play a role in a number of physiological activities such as homeostatic regulation of the immune system, elimination of invading pathogens and clearance of apoptotic or cancer cells (Holodick et al., 2017). Several lines of evidence suggest that natural autoantibodies are associated with some common chronic illnesses including type-2 diabetes, atherosclerosis, and malignant tumor (Cai et al., 2017; Wang et al., 2018; Zhao et al., 2018; Liu et al., 2020). In fact, anti-tumor cytotoxicity of natural autoantibodies has been confirmed with *in vitro* study and catches more attention lately. In a previous study, we found that healthy individuals had remarkably high levels of natural IgG antibodies against human vascular endothelial growth factor receptor 1 (VEGFR1) and that anti-VEGFR1 IgG-abundant plasma could inhibit the proliferation of liver cancer cells (Wang et al., 2017). In this study, therefore, we investigated circulating levels of natural autoantibodies against HER2 in OSCC patients and analyzed the effects of anti-HER2 IgG-abundant plasma and its intravenous immunoglobulin (IVIg) on OSCC cells.

## Materials and Methods

### Participants

A total of 88 patients with OSCC and 105 patients with oral benign tumor, who were admitted to the Beijing Stomatological Hospital of Capital Medical University in the period between December 2018 and August 2020, were recruited for this study; 120 healthy subjects were simultaneously recruited as controls from local communities. The demographic and clinical information was given in Table 1. All patients with OSCC underwent histological confirmation for their diagnosis and tumor stages; their plasma samples were obtained after diagnosis was made but prior to any anticancer treatment given. Patients with oral benign tumor and OSCC experienced clinical interview and radiographic or imaging examination to exclude those who had any other malignancy or autoimmune diseases. To explore whether plasma anti-HER2 IgG levels were altered in different stages of OSCC, these patients were divided into two subgroups based on the TNM (tumor, node, and metastasis) staging system: early stage (Tis + T1N0M0 + T2N0M0), and middle & advanced stage (stages 3 and 4). All the participants provided written informed consent to participate in the study as approved by the Research Ethics Board of the Beijing Stomatological Hospital of Capital Medical University (Approval code: 2015-92 and 2019-126) and conformed to the Declaration of Helsinki.

**TABLE 1:** Demographic and clinical characteristics of control, benign-tumor and OSCC subjects.

Characteristics	Control (*n* = 120)	Benign (*n* = 105)	OSCC (*n* = 88)	*p* ^a^	*p* ^b^
Gender, *n* (%)					
Male	83 (69.2)	52 (49.5)	50 (56.8)	0.067	0.312
Female	37 (30.8)	53 (50.5)	38 (43.2)		
Age ( $\bar{X}$ ± SD)	50.2 ± 2.2	46.5 ± 14.9	58.9 ± 11.0	<0.001	<0.001
TNM subgroups (%)					
Early stage	—	—	39 (44.3)		
Middle & advanced stage	—	—	49 (55.7)		

a Control vs. OSCC.

b Benign vs. OSCC.

### Detection of Plasma Anti-HER2 IgG Levels

Linear peptide antigens derived from the extracellular domain of human HER2 protein (NP_004439.2) were designed using a computational epitope prediction software (http://www.iedb.org) based on the features of the target sequences such as hydrophilicity, flexibility, surface accessibility and antigenicity; they were then synthesized by solid-phase chemistry with a purity of >95%. An enzyme-linked immunosorbent assay (ELISA) was developed in-house as described previously (Wang et al., 2017; Zhao et al., 2018; Liu et al., 2020). A total of 200 plasma samples from healthy blood donors were screened at the Blood Center of Dongguan, Guangdong Province, China. To reduce inter-plate deviation, pooled plasma from >100 randomly selected individuals was used as a quality control (QC) sample to assess reproducibility of the in-house ELISA for relative quantification of plasma anti-HER2 IgG levels. All the ELISA reagents were provided by Hailanshen Biotechnology Ltd., Qingdao, China. Negative control (NC) was PBS-based assay buffer and positive control (PC) was purified IgG from human blood (G4386, Sigma-Aldrich, United States). The optical density (OD) was measured on a microplate reader at 450 nm with a reference wavelength of 620 nm. All the samples were tested in duplicate and specific binding ratio (SBR) was used to represent plasma anti-HER2 IgG levels. The SBR is calculated as follows:

$$SBR=\frac{OD_{sample}-OD_{NC}}{OD_{PC}-OD_{NC}}$$

### Histological Assay

OSCC and cancer-adjacent normal tissues were fixed with 10% (v/v) neutral formaldehyde, and then processed routinely for paraffin embedding, preparation of tissue sections with 4 μm serial sections, followed by deparaffinization. The slices were used for Hematoxylin-eosin (H&E) staining and immunohistochemistry (IHC) analysis. After routine deparaffinization and rehydration, the paraffin-embedded tissue slices were heated in a pressure cooker containing 10 mM citrate buffer (pH 6.0) for 10 min to repair the antigen and the endogenous peroxidase was then quenched by 3% H_2_O_2_. After blocking, the slices were incubated with the anti-HER2 antibody working solution (A2071, Abclonal, Wuhan, China) overnight at 4°C, and the secondary antibody (Maxim Biotechnologies, Beijing, China) conjugated with HRP was then incubated and stained with 3,3′-diaminobenzidine (DAB) for observation. Hematoxylin was used for a counterstain. H&E and IHC staining image acquisition was performed under the Olympus BX61 microscope (Olympus, Tokyo, Japan); the average optical density (AOD) was counted using ImageProPlus 6.0 software.

### Cell Culture

Three OSCC-derived cell lines, CAL27, SCC25, and SCC15 (ATCC, United States), were cultured in Dulbecco’s Modified Eagle Medium (DMEM, Gibco, NY, United States) and RPMI 1640 Medium (Gibco), respectively, and both media contained 10% fetal bovine serum (FBS, Gibco). All cell lines were cultured in humidified atmosphere with 5% CO_2_ at 37°C.

### Cell Proliferation Assay

Boya Bio-pharmaceutical Group Co., Ltd., China kindly provided anti-HER2 IVIg that was extracted from anti-HER2 IgG-abundant plasma and regular IVIg that was extracted from anti-HER2 IgG-deficient plasma. Cell proliferation was analyzed using the Cell Counting Kit-8 (CCK-8) reagent (Dojindo, Tokyo, Japan), and 5-ethynyl-2′-deoxyuridine (EdU) staining according to the manufacturer’s instructions. Briefly, OSCC cells were seeded in a 96-well plate and pre-incubated for 24 h with complete medium containing 10% FBS. These cells were then treated with following conditions for 48 h: 20% anti-HER2 IgG-abundant plasma only, 20% anti-HER2 IgG-deficient plasma only, 20% FBS with trastuzumab (Roche, Genentech, Inc. CA, United States), 20% anti-HER2 IgG-deficient plasma plus 200 μg/ml trastuzumab or plus regular IVIg (2.5 mg/ml or 5 mg/ml) or plus anti-HER2 IVIg (2.5 mg/ml or 5 mg/ml), respectively. Cell viability was used to present data and calculated based on the CCK-8 OD signal as follows:

$$Cell\;Viability=\frac{OD_{abundant}-OD_{blank}}{OD_{deficient}-OD_{blank}}$$

EdU staining was conducted using BeyoClick™ EdU Cell Proliferation Kit with Alexa Fluor 555 (Beyotime, Shanghai, China) according to the manufacturer’s protocol. The treated OSCC cells were incubated with EdU working solution (10 µM) in the dark at 37°C for 2 h, and then fixed with 4% paraformaldehyde for 15 min. Next, the cells were permeabilized with 0.1% Triton X100 for 15 min, and then incubated with Hoechst 33342 for 5 min. The image was captured with a fluorescence microscope, and the image was taken at 200× magnification (Olympus, Tokyo, Japan) to calculate the rate of cell proliferation. OSCC cells that underwent DNA replication during the incubation showed red fluorescence, while the nucleus showed blue fluorescence.

### Analysis of Apoptosis

The percentage of OSCC cell lines in early and late apoptosis was determined by AnnexinV-FITC/ propidium iodide (PI) staining according to the instruction (BD Biosciences, United States). The cells were then analyzed through FlowJo V10 software. Apoptotic cells were detected by FITC-Annexin V staining, while PI staining was used to discriminate apoptotic, dead, and necrotic cells. Annexin V-FITC+/PI− and Annexin V-FITC+/PI+ staining was used to define early and late apoptosis.

### Transwell Invasion Assay

A conventional 24-well transwell invasion system (Corning, NY, United States) was applied to analyze the ability of cell invasion/metastasis. 5 × 10^5^ OSCC cells in serum-free medium was seeded in triplicate on the top chamber coated with Matrigel (100 µl per well, thickness: 3 mm; Becton Dickinson, San Jose, CA, United States), and then incubated for 48 h in medium containing 20% FBS added to the lower chamber as a driving factor. OSCC cells on the lower surface of the membrane were fixed with 4% formalin and stained with Giemsa (Salarbio, Beijing, China). The polycarbonate membrane was cut and mounted on the slide for preservation. Images of the cells attached to the undersurface of the membrane were captured with 200× magnification microscope (Olympus BX61).

### Western Blotting Assay

Cultured OSCC cells were harvested and lysed in cell lysis reagent (Sigma-Aldrich, St. Louis, United States) to extract total cell protein. Equal amounts of proteins were separated by sodium dodecyl sulfate (SDS) polyacrylamide gel electrophoresis on 4–15% gels (Bio-Rad, CA, United States) and then electrophoretically transferred to polyvinylidene difluoride membranes (Bio-Rad). The membranes were blocked at room temperature and incubated overnight at 4°C with the appropriate primary antibody; HRP-conjugated goat anti-rabbit IgG (AS014, ABclonal) was then added and incubated. Following extensive washing, the immune-reactive proteins on the membrane were visualized with Clarity Western ECL Substrate (1705060, Bio-Rad) and measured *via* computerized image analysis (ChemiDoc MP, Bio-Rad). All primary antibodies used are listed in Supplementary Table S1.

### Quantitative Real-Time PCR

Total RNA was isolated from cultured OSCC cell lysates using the Trizol Reagent (Invitrogen, Carlsbad, United States), and Nanodrop spectrophotometer (California Santa Clara, Agilent Technologies, CA, United States) was used to measure the concentrations of RNA samples. The SuperRT cDNA synthesis kit (CWbio, Beijing, China) was used to generate cDNA, and the UltraSYBR one-step qPCR kit (Low ROX, CWbio) was used to quantify the expression of target genes. Glyceraldehyde 3-phosphate dehydrogenase (*GAPDH*) was used as a housekeeping gene for normalization, and the 2^−△△CT^ method was used to quantify relative levels of gene expression. The primary sequences used for qPCR amplification are given as follows: *HER2*, 5′- GAC​TGC​CTG​TCC​CTA​CAA​T-3′ (forward) and 5′-TCC​TCT​GCT​GTC​ACC​TCT​TG-3′ (reverse); *GAPDH*, 5′-AGG​TCG​GTG​TGA​ACG​GAT​TTG-3′ (forward) and 5′-TGT​AGA​CCA​TGT​AGT​TGA​GGT​CA-3′ (reverse).

### siRNA Transfection

All siRNAs used in this study were synthesized by GenePharma (Shanghai, China). Before siRNA transfection, OSCC cells were seeded in 6-well plates and cultured for 24 h. These cells were then transfected with siRNA Oligo (100 pmol/well) against GP-transfect-Mate (GenePharma) diluted in serum-free DMEM medium according to the manufacturer’s instructions; the efficiency of knockdown was determined by qPCR and Western blot 24 and 48 h after siRNA transfection. The siRNA sequences were listed in Supplementary Table S2.

### Statistical Analysis

The coefficient of variation (CV) was used to represent an inter-assay deviation estimated using the QC sample tested on every 96-well plate as mentioned above. Plasma IgG levels were expressed as the mean ± standard deviation (SD) in SBR. Because Kolmogorov–Smirnov one-sample test showed a skewed distribution of plasma IgG levels in all three groups (Supplementary Table S3), Kruskal-Wallis H test was applied to examine their differences. Binary regression analysis was applied to examine the differences in plasma anti-HER2 IgG levels between OSCC patients and control subjects or between OSCC patients and benign-tumor patients, with adjustment for gender and age. Student’s *t*-test (two-tailed) and one-way ANOVA were also applied to analyze the experimental data. *p* < 0.05 was considered to be statistically significant.

## Results

### HER2 Expression in OSCC Cancer Tissues and Adjacent Tissues

**FIGURE 1:**
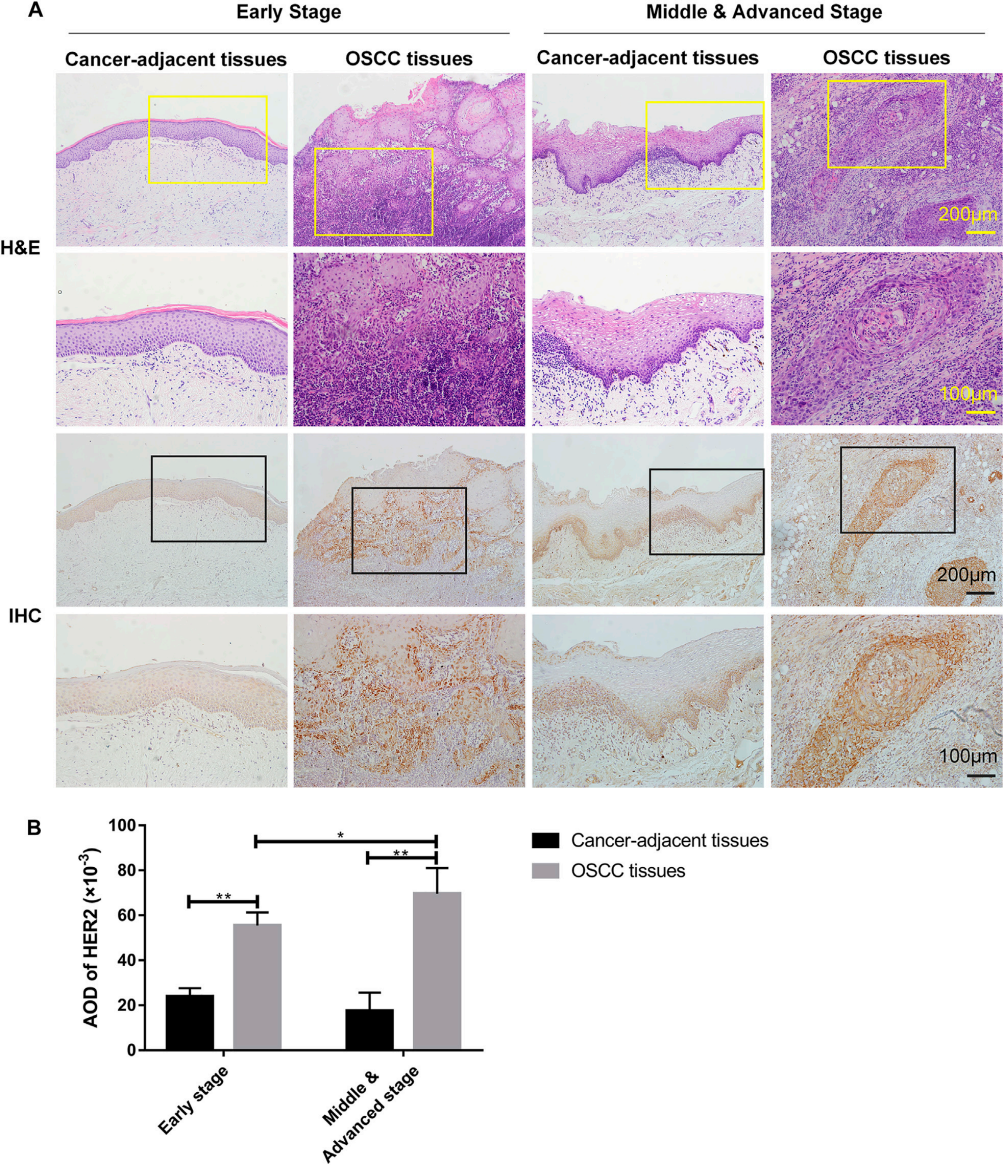
The expression of HER2 in OSCC tissues. **(A)** H&E and IHC staining of OSCC tissues and cancer-adjacent tissues in early (*n* = 5), middle & advanced stages (*n* = 5). **(B)** The average optical density (AOD) data of IHC staining were presented as mean ± standard deviation (SD). *p < 0.05; **p < 0.01.

Following pathological diagnosis of clinical cases, we randomly collected 10 individual samples of cancer and adjacent tissues from OSCC patients in early stage and middle & advanced stage, respectively, for H&E and IHC staining, and then analyzed the relationship between HER2 expression and tumor progression. H&E staining showed normal epithelial features in all cancer-adjacent tissues as compared with OSCC tissues. The cancer cells were arranged to form solid nests or strands as well as infiltrative pattern growth; the nuclear pleomorphism of cancer cells was obviously increased with abnormal mitotic nuclear (Figure 1A). The IHC staining showed higher expression of HER2 in OSCC tissues than normal adjacent tissues (*p* = 6.89 × 10^−6^ for early stage and *p* = 3.26 × 10^−5^ for middle & advanced stage), and also in cancer tissues from middle & advanced than early stage OSCC (*p* = 0.038) (Figure 1B), demonstrating that the level of HER2 expression was gradually increased with the progression of OSCC.

### Changes of Plasma Anti-HER2 IgG Levels in Patients With OSCC

The in-house ELISA developed showed a good reproducibility with a CV of 2.75% based on the anti-HER2 IgG assay with QC plasma (Supplementary Table S4). As shown in Table 2, plasma anti-HER2 IgG levels were significantly different between control subjects, oral benign tumor and OSCC patients (*H* = 11.820, *p* = 0.003). Further analysis was performed to compare plasma anti-HER2 IgG levels between subgroups and indicated that anti-HER2 IgG levels were slightly lower in patients with oral benign tumor and early stage OSCC than control subjects, while plasma anti-HER2 IgG levels showed a trend toward an increase in OSCC patients at the middle & advanced stages compared with those at the early stage (Table 2). Because the age distribution was significantly different between these three study groups (Table 1), a binary logistic regression analysis was applied to examine the differences in plasma IgG levels with adjustment for age and gender but failed to show a difference among these three groups, suggesting that this alteration might be caused by the bias in age distribution (Supplementary Table S5).

**TABLE 2:** The levels of plasma IgG against HER2 among three different groups.

Group	Control (n)	Benign (n)	OSCC (n)	*H* ^a^	*p* ^b^
TMN Subgroups^c^					
Early stage	0.32 ± 0.10 (120)	0.27 ± 0.11 (105)	0.31 ± 0.12 (39)	11.91	0.003
Middle & advanced stage	0.32 ± 0.10 (120)	0.27 ± 0.11 (105)	0.33 ± 0.16 (49)	12.49	0.002
Total	0.32 ± 0.10 (120)	0.27 ± 0.11 (105)	0.32 ± 0.14 (88)	11.82	0.003

Plasma anti-HER2 IgG levels were expressed as mean ± SD in SBR.

a Kruskal-Wallis *H* test.

b *p* < 0.05 was considered statistically significant as three individual groups were tested.

c Early stage was defined as stages Tis + T1N0M0 + T2N0M0, and middle & advanced stages were defined as stages 3 and 4.

### The Role of Anti-HER2 IgG-Abundant Plasma in Inhibiting OSCC by Induction of Cell Apoptosis

**FIGURE 2:**
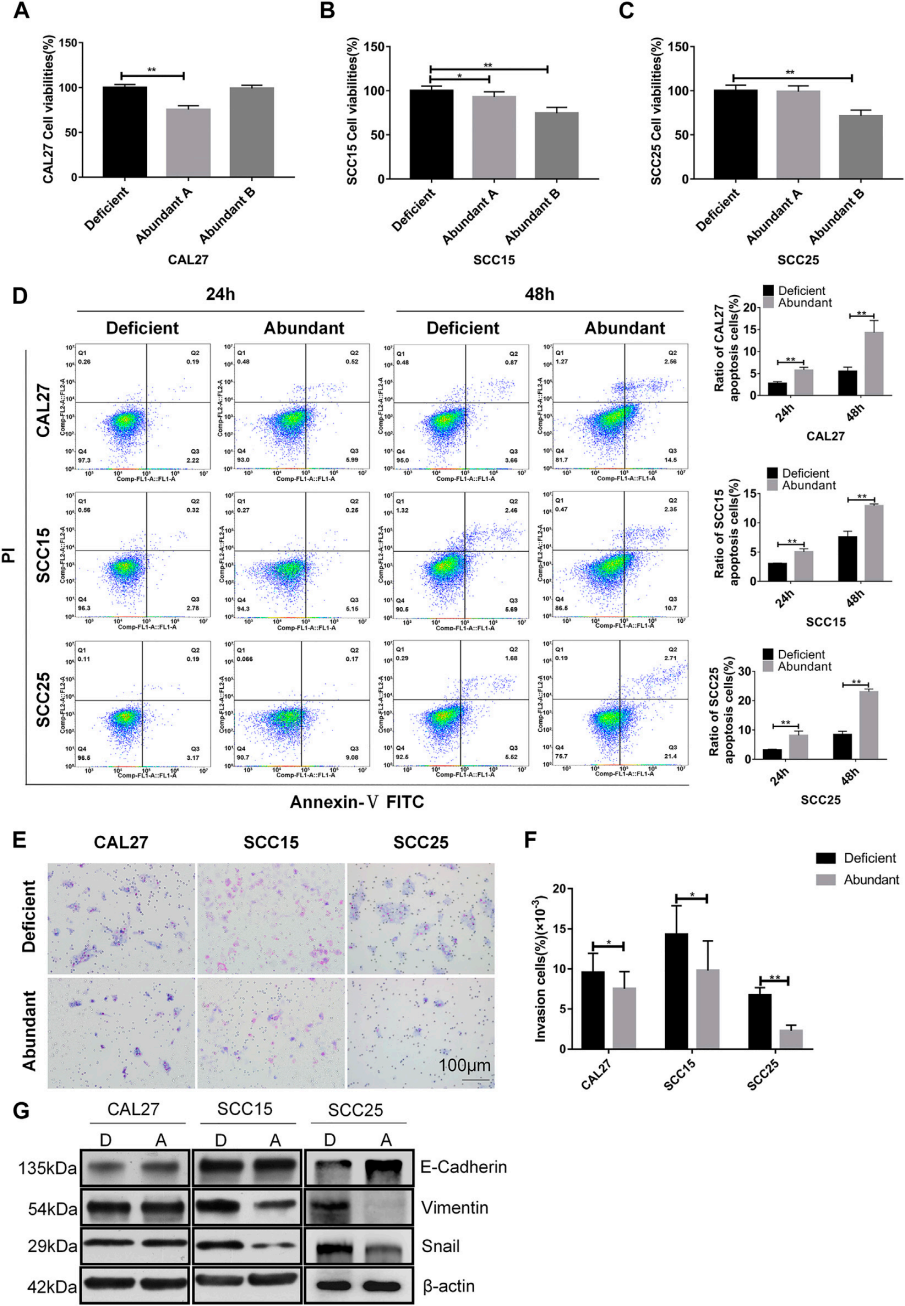
Effects of anti-HER2 IgG-abundant plasma on the proliferation of OSCC cells. **(A–C)** Proliferation of three OSCC cell lines treated with anti-HER2 IgG-abundant plasma for 48 h. Plasma A and plasma B obtained from two individual healthy donors were abundant in anti-HER2 IgG. The data of proliferation were expressed as mean ± SD in cell viability (%). Plasma A showed an inhibitory effect on CAL27 and plasma B on both SCC15 and SCC25 cells. **(D)** Apoptosis of three OSCC cell lines treated with anti-HER2 IgG-abundant and -deficient plasma for 24 and 48 h, respectively. The data were expressed as mean ± SD in proportion of apoptotic cells. **(E–F)** Three OSCC cell lines treated with either 20% anti-HER2 IgG-abundant or -deficient plasma for 48 h were photographed and bar chart showed the proportion of invasive cells relative to total number of seeding cells. **(G)** Three OSCC cell lines were cultured in 20% anti-HER2 IgG-abundant or -deficient plasma and the expression of EMT-related factors, E-cadherin, Vimentin, or Snail was then determined by Western blot with β-actin as reference. D: anti-HER2 IgG-deficient plasma; A: anti-HER2 IgG-abundant plasma; *p < 0.05; **p < 0.01.

According to the analysis of plasma samples showing an increase in natural anti-HER2 IgG levels with the severity of tumors, we screened 200 plasma samples from healthy donors by the in-house ELISA to identify anti-HER2 IgG-abundant plasma; two samples with the highest anti-HER2 IgG levels were selected as the anti-HER2 IgG-abundant plasma (assigned A and B, respectively) and pooled plasma from six individual donors with the lowest anti-HER2 IgG levels was used as the anti-HER2 IgG-deficient plasma for baseline signals. Three pre-incubated OSCC cell lines, CAL27, SCC15, and SCC25 cells, were cultured in medium containing either 20% anti-HER2 IgG-deficient or 20% anti-HER2 IgG-abundant plasma for 48 h. The CCK-8 assay showed that anti-HER2 IgG-abundant plasma could significantly inhibit the proliferation of CAL27 cells treated with plasma A (*p* = 4.25 × 10^−11^), and SCC15 and SCC25 cells treated with plasma B (*p* = 2.02 × 10^−8^ for SCC15 cells and *p* = 5.2 × 10^−8^ for SCC25 cells) (Figures 2A–C). To address the mechanism behind the inhibitory effect of anti-HER2 IgG-abundant plasma on OSCC cells, we investigated cell apoptosis induced by 20% anti-HER2 IgG-abundant plasma. As shown in Figure 2D, the proportion of apoptotic cells was significantly increased in CAL27 cells (*p* = 0.0019 for 24 h treatment and *p* = 0.0063 for 48 h treatment), SCC15 cells (*p* = 0.0024 for 24 h treatment and *p* = 0.0009 for 48 h treatment), and SCC25 cells (*p* = 0.0046 for 24 h treatment and *p* = 6.29 × 10^−5^ for 48 h treatment). Transwell invasion assay demonstrated that anti-HER2 IgG-abundant plasma could inhibit the invasion of CAL27 cells (*p* = 0.021), SCC15 cells (*p* = 0.013), and SCC25 cells (*p* = 7.87 × 10^−15^) (Figures 2E,F). The expression of epithelial-mesenchymal transition (EMT)-related biomarkers was examined. As shown in Figure 2G, the expression of E-Cadherin was up-regulated in CAL27 and SCC25 cells treated with anti-HER2 IgG-abundant plasma, while the expression of Vimentin and Snail was down-regulated in SCC15 and SCC25 cells, suggesting that anti-HER2 IgG-abundant plasma could significantly inhibit cell proliferation and invasion, and promote the apoptosis of OSCC cells.

### Possible Involvement of the CDC Pathway

**FIGURE 3:**
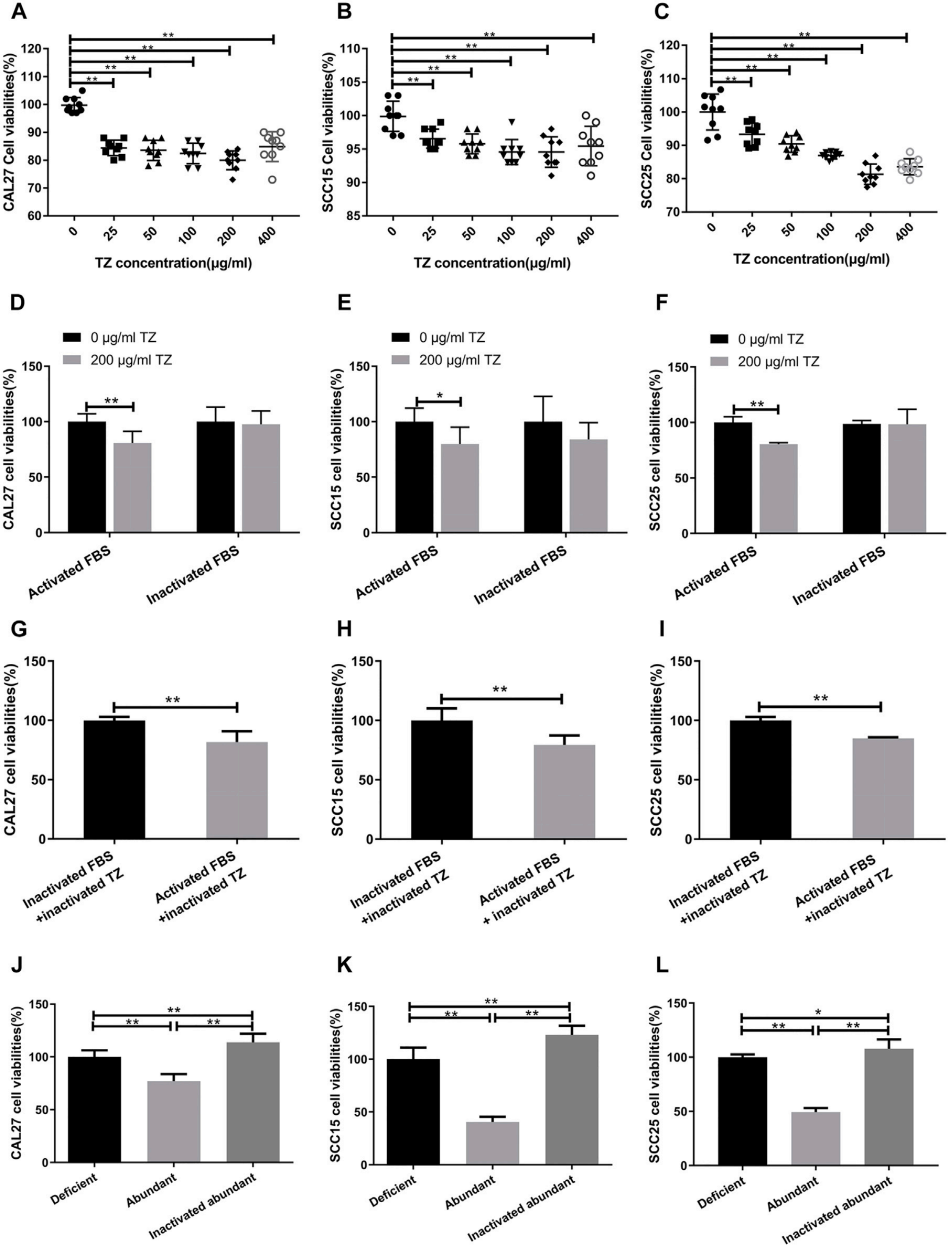
Possible involvement of complement-dependent cytotoxicity. **(A–C)** Inhibitory effects of trastuzumab (TZ) on proliferation of CAL27, SCC15, and SCC25 cells. A range of the TZ concentrations was used to treat OSCC cells and the concentration of 200 μg/ml TZ was considered as the optimum concentration for subsequent experiments. **(D–F)** Viability of CAL27, SCC15, and SCC25 cells treated with 200 μg/ml TZ in medium containing either 20% inactivated FBS or 20% activated FBS. **(G–I)** Viability of CAL27, SCC15, and SCC25 cells treated with inactivated 200 μg/ml TZ in medium containing either 20% inactivated FBS or 20% activated FBS. **(J–L)** Viability of CAL27, SCC15, and SCC25 cells treated with 20% anti-HER2 IgG-deficient plasma, 20% anti-HER2 IgG-abundant plasma and 20% inactivated anti-HER2 IgG-abundant plasma. The data of proliferation were expressed as mean ± SD in cell viability (%). Deficient, anti-HER2 IgG-deficient plasma; Abundant, anti-HER2 IgG-abundant plasma; TZ, trastuzumab; *p < 0.05; **p < 0.01.

The complement-dependent cytotoxicity (CDC) pathway has been considered to play a critical role in anti-tumor effect of mAbs. Based on a dose-dependent curve from trastuzumab-treated OSCC cells for 48 h at the concentrations of 0, 25, 50, 100, 200 and 400 μg/ml, respectively, 200 μg/ml trastuzumab in a medium containing 20% fetal bovine serum (FBS) appeared to be the optimal concentration of inhibiting OSCC proliferation (*p* = 3.18 × 10^−10^ for CAL27 cells, *p* = 1.41 × 10^−4^ for SCC15 cells, and *p* = 1.1 × 10^−7^ for SCC25 cells) as compared with 0 μg/ml trastuzumab treatment (Figures 3A–C). When FBS was inactivated at 56°C for 30 min to destroy the complement system, trastuzumab no longer showed the inhibitory effect on OSCC cell proliferation (*p* = 0.0003 for CAL27 cells, *p* = 0.006 for SCC15 cells, and *p* = 6.3 × 10^−9^ for SCC25 cells) (Figures 3D–F). When trastuzumab was also heated at 56°C for 30 min, this mAb appeared to be stable and still had inhibitory effect on the growth of OSCC cells (*p* = 2.91 × 10^−5^ for CAL27 cells, *p* = 1.98 × 10^−4^ for SCC15 cells, and *p* = 1.26 × 10^−10^ for SCC25 cells) (Figures 3G–I). To confirm if anti-HER2 IgG-abundant plasma inhibited OSCC cell proliferation via the CDC pathway, heat-inactivated anti-HER2 IgG-abundant plasma was used to replicate the above finding. The results demonstrated that OSCC cells grew more quickly in medium containing 20% inactivated plasma than 20% normal plasma (*p* = 1.39 × 10^−8^ for CAL27 cells, *p* = 3.29 × 10^−14^ for SCC15 cells and *p* = 3.02 × 10^−16^ for SCC25 cells) (Figures 3J–L), suggesting that anti-tumor effects of natural anti-HER2 IgG autoantibodies were mediated through the CDC pathway. Since trastuzumab was mainly applied to treat patients with breast cancer that showed high expression of the HER2 gene, we performed an *in vitro* study to compare the difference in anti-tumor effects between anti-HER2 IgG-abundant plasma and trastuzumab on two breast cancer-derived cell lines, SK-BR-3 and BT-474, which highly express HER2 (Supplementary Materials and Methods). When SK-BR-3 and BT-474 cells were cultured in medium containing 20% anti-HER2 IgG-abundant plasma only and in medium containing 20% anti-HER2 IgG-deficient plasma and 200 μg/ml trastuzumab, respectively, trastuzumab could significantly inhibit the proliferation of BT-474 cells (*p* = 1.49 × 10^−7^) but not SK-BR-3 cells (Supplementary Figures S1A,B), whereas anti-HER2 IgG-abundant plasma could significantly inhibit the proliferation of both breast cancer cell lines (*p* = 0.006 for SK-BR-3 cells and *p* = 1.71 × 10^−7^ for BT-474 cells). Interestingly, the inhibitory effect on proliferation of breast cancer cells disappeared after heat-inactivated anti-HER2 IgG-abundant plasma was applied.

### Inhibitory Effect of Anti-HER2 IVIg on OSCC Cell Proliferation

**FIGURE 4:**
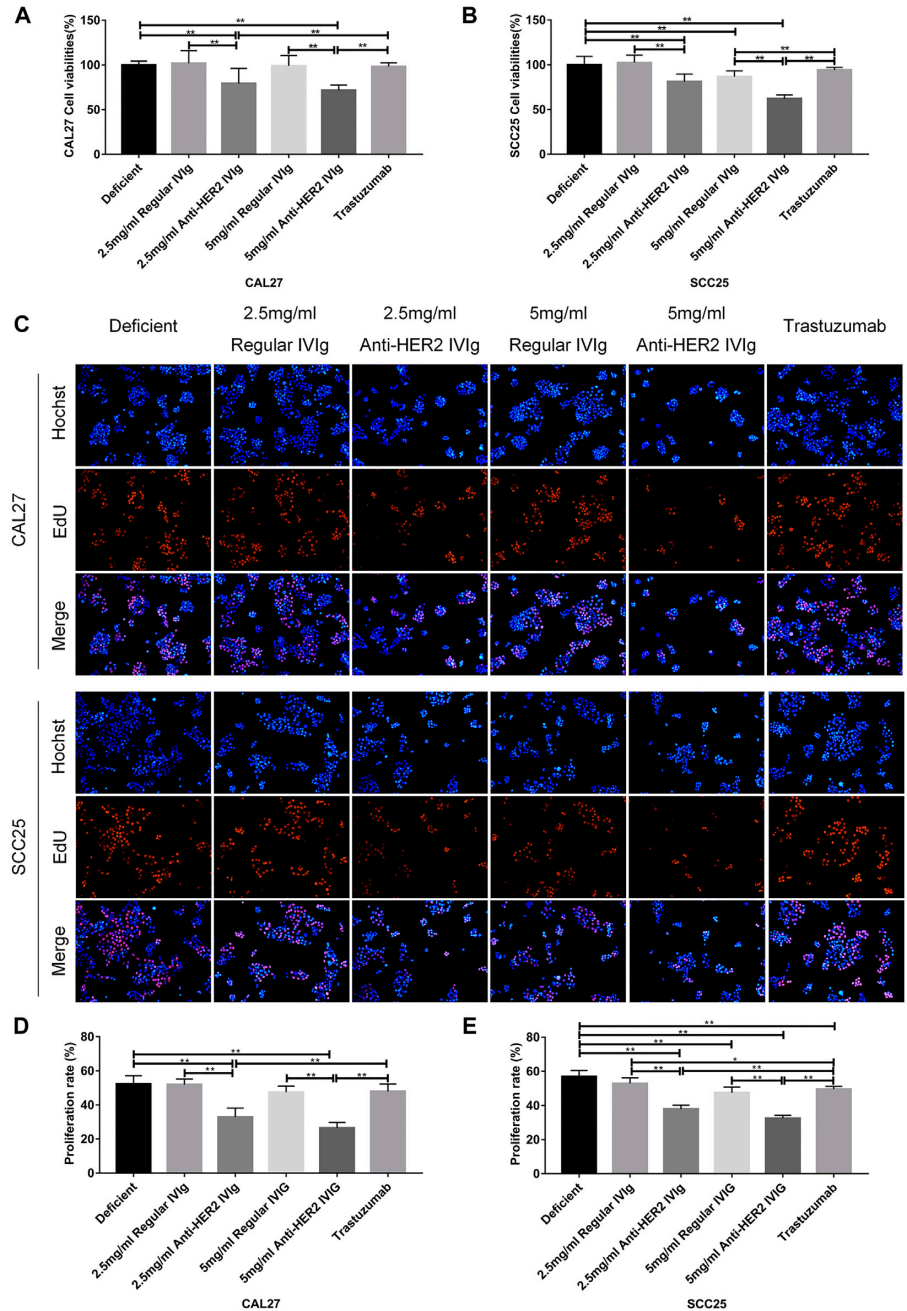
Inhibitory effect of anti-HER2 IVIg on the proliferation of OSCC cells. **(A,B)** Viability of CAL27 cells and SCC25cells treated with anti-HER2 IgG-deficient plasma only, anti-HER2 IgG-deficient plasma containing 200 μg/ml trastuzumab, 2.5 mg/ml regular IVIg, 2.5 mg/ml anti-HER2 IVIg, 5 mg/ml regular IVIg, and 5 mg/ml anti-HER2 IVIg, respectively. **(C–E)** EdU staining of CAL27 and SCC25 cells treated with anti-HER2 IgG-deficient plasma only, 200 μg/ml trastuzumab, 2.5 mg/ml regular IVIg, 2.5 mg/ml anti-HER2 IVIg, 5 mg/ml regular IVIg, and 5 mg/ml anti-HER2 IVIg, in medium containing 20% anti-HER2 IgG-deficient plasma. The data were expressed as mean ± SD in cell viability (%). Deficient, anti-HER2 IgG-deficient plasma; IVIg, intravenous immunoglobulin; *p < 0.05; **p < 0.01.

To rule out the effect of other components in plasma on cancer cells, both anti-HER2 IgG-abundant and -deficient plasma were used to prepare for anti-HER2 IVIg and regular IVIg, respectively, and to conduct the following experiments with CAL27 and SCC25 cell lines (Supplementary Figure S2). According to the physiological IgG levels in human blood, the concentrations of 2.5 and 5 mg/ml IVIg in 20% anti-HER2 IgG-deficient plasma were chosen to treat CAL27 and SCC25 cells for 48 h with the following conditions: 20% anti-HER2 IgG-deficient plasma only, 20% anti-HER2 IgG-deficient plasma containing 200 μg/ml trastuzumab, and 20% anti-HER2 IgG-deficient plasma containing regular IVIg or anti-HER2 IVIg. The CCK-8 assay showed that both 2.5 mg/ml IVIg and 5 mg/ml anti-HER2 IVIg could significantly inhibit CAL27 cell proliferation (*p* = 0.007 for 2.5 mg/ml treatment and *p* = 0.0005 for 5 mg/ml treatment) and SCC25 cell proliferation (*p* = 7.84 × 10^−5^ for 2.5 mg/ml treatment and *p* = 4.63 × 10^−8^ for 5 mg/ml treatment), as compared with the regular IVIg. Intriguingly, 200 μg/ml trastuzumab had no effect on OSCC proliferation (Figures 4A,B). Meanwhile, EdU staining further verified the above results (Figures 4C–E), although the anti-HER2 IVIg treatment showed a significant inhibitory effect on two breast cancer cell lines (*p* = 6.33 × 10^−7^ for SK-BR-3 cells and *p* = 1.08 × 10^−8^ for BT-474 cells), as compared with regular IVIg treatment (Supplementary Figures S1C,D). It is worth noting that trastuzumab could also inhibit the proliferation of two breast cancer cell lines but its inhibitory intensity was much weaker than anti-HER2 IVIg (*p* = 3.05 × 10^−6^ for SK-BR-3 cells and *p* = 2.32 × 10^−10^ for BT-474 cells).

### Induction of OSCC Cell Apoptosis by Anti-HER2 IVIg

**FIGURE 5:**
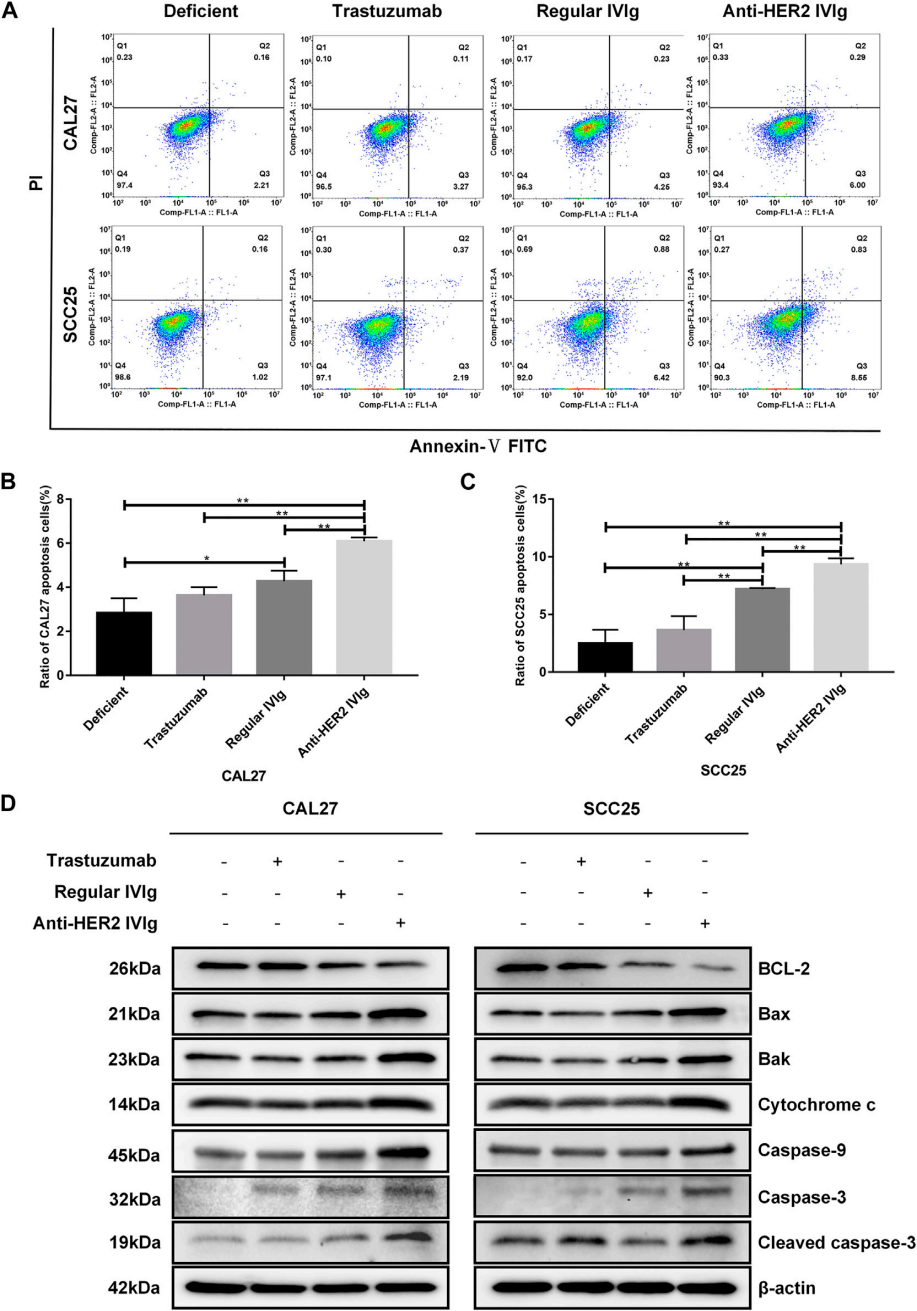
The effects of anti-HER2 IVIg on OSCC cells apoptosis. **(A–C)** Apoptosis of CAL27 and SCC25 cells treated for 48 h with anti-HER2 IgG-deficient plasma only, 200 μg/ml trastuzumab, 5 mg/ml regular IVIg, and 5 mg/ml anti-HER2 IVIg, respectively. The data were expressed as mean ± SD in proportion of apoptotic cells. **(D)** CAL27 cells and SCC25 cells were treated for 48 h with anti-HER2 IgG-deficient plasma only, 200 μg/ml trastuzumab, 5 mg/ml regular IVIg, and 5 mg/ml anti-HER2 IVIg, respectively; the expression of apoptosis-related factors, including Bcl-2, Bax, Bak, cytochrome c, caspase-9, caspase-3, and cleaved caspase-3, was then determined by Western blot with β-actin as a reference. Deficient, anti-HER2 IgG-deficient plasma; IVIg, intravenous immunoglobulin; *p < 0.05; **p < 0.01.

To explore the mechanism by which anti-HER2 IVIg could significantly inhibit OSCC cell proliferation, cell apoptosis was examined in OSCC cells treated with anti-HER2 IVIg, regular IVIg, or trastuzumab in subsequent experiments. As shown in Figures 5A–C, the proportion of apoptotic OSCC cells was significantly increased in the 5 mg/ml anti-HER2 IVIg treatment compared with 200 μg/ml trastuzumab treatment (*p* = 0.0036 for CAL27 cells and *p* = 0.002 for SCC25 cells). In addition, regular IVIg treatment could also induce the apoptosis of OSCC cells with a weaker effect than anti-HER2 IVIg treatment (*p* = 0.00048 for CAL27 cells and *p* = 0.0017 for SCC25 cells) (Figures 5A–C). Western blot assay showed that Bcl-2 expression was significantly down-regulated in both CAL27 and SCC25 cells treated with anti-HER2 IVIg, while the expressions of Bax, Bak, cytochrome c, casepase-9, casepase-3, and cleaved casepase-3 were significantly up-regulated, compared with regular IVIg treatment (Figure 5D). However, the expression of cleaved casepase-3 was slightly increased only in SCC25 cells treated with 200 μg/ml trastuzumab.

### Inhibitory Effect of Anti-HER2 IVIg on OSCC Cell Invasion

**FIGURE 6:**
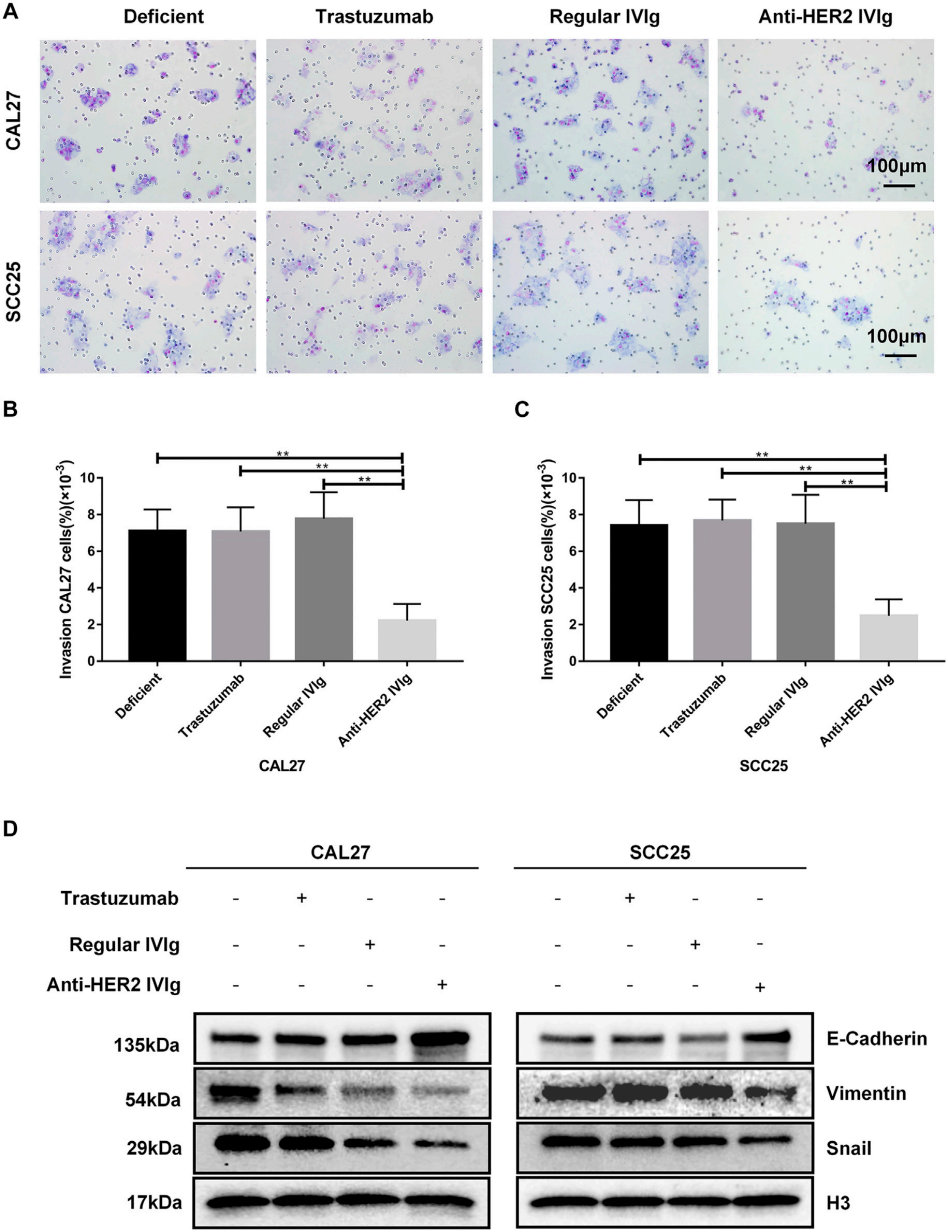
Inhibitory effects of anti-HER2 IVIg on OSCC cells migration. **(A–C)** CAL27 and SCC25 cells treated for 48 h with anti-HER2 IgG-deficient plasma only, 200 μg/ml trastuzumab, 5 mg/ml regular IVIg, and 5 mg/ml anti-HER2 IVIg, respectively, were photographed; bar chart showed the proportion of invasive cells relative to the total number of seeding cells. **(D)** CAL27 and SCC25 cells were cultured for 48 h with anti-HER2 IgG-deficient plasma only, 200 μg/ml trastuzumab, 5 mg/ml regular IVIg, and 5 mg/ml anti-HER2 IVIg, respectively; the expression of EMT-related factors, E-cadherin, Vimentin, or Snail was then determined by Western blot with H3 as a reference. Deficient: anti-HER2 IgG-deficient plasma; IVIg: intravenous immunoglobulin; *p < 0.05; **p < 0.01.

OSCC is prone to recurrence and metastasis, which possibly is the main reason for the poor prognosis of this malignancy. Therefore, transwell assay was applied to explore the inhibitory effect of anti-HER2 IVIg on OSCC invasion. The results demonstrated that 5 mg/ml anti-HER2 IVIg treatment could inhibit CAL27 cell invasion (*p* = 5.15 × 10^−13^) and SCC25 cell invasion (*p* = 1.86 × 10^−11^) compared with regular IVIg; the invasion ability was about 4 times lower than regular IVIg treatment. However, 200 μg/ml trastuzumab failed to show inhibition of OSCC invasion (Figures 6A–C). Western blotting assay demonstrated that E-cadherin expression was up-regulated and the expression of Vimentin and Snail was down-regulated in CAL27 and SCC25 cells treated with anti-HER2 IVIg compared with those treated with trastuzumab and regular IVIg (Figure 6D).

### Effects of Anti-HER2 IVIg on Proliferation and Apoptosis of HER2-Knockdown OSCC Cells

**FIGURE 7:**
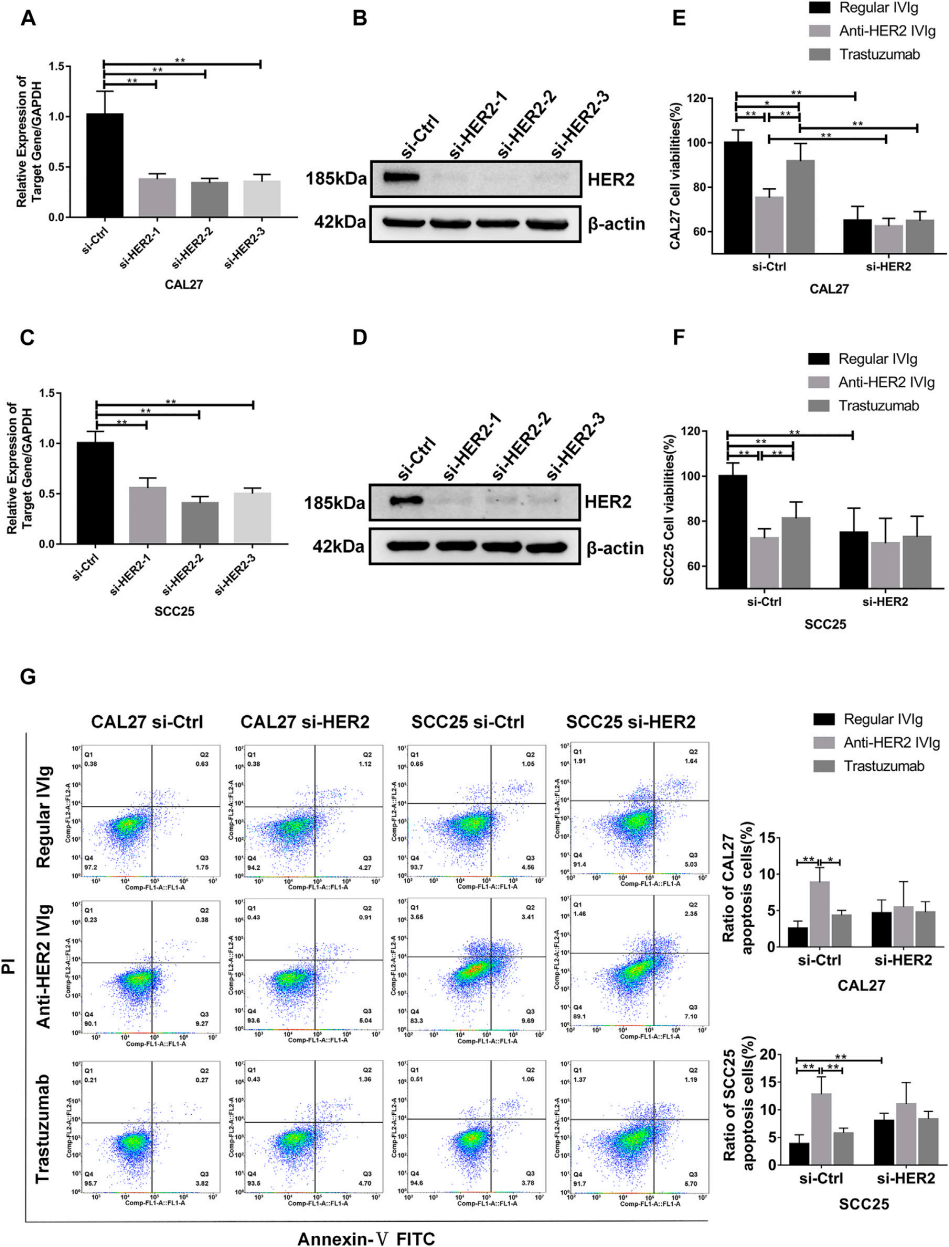
Inhibitory effects of anti-HER2 IVIg on HER2 knockdown OSCC cells. **(A–D)** Three HER2-specific siRNAs were transfected into CAL27 and SCC25 cells. The expression of HER2 mRNA and protein was then examined by qPCR and Western blot assay, respectively. **(E–F)** CAL27 and SCC25 cells were transfected with either HER2-specific siRNAs or control siRNAs; OSCC cells were treated for 48 h with anti-HER2 IgG-deficient plasma containing 200 μg/ml trastuzumab, 5 mg/ml regular IVIg, and 5 mg/ml anti-HER2 IVIg, respectively. The data were expressed as mean ± SD in cell viability (%). **(G)** CAL27 and SCC25 cells were transfected with either HER2-specific siRNAs or control siRNAs, and then treated for 48 h with anti-HER2 IgG-deficient plasma containing 200 μg/ml trastuzumab, 5 mg/ml regular IVIg, and 5 mg/ml anti-HER2 IVIg, respectively. The data were expressed as mean ± SD in proportion of apoptotic cells. IVIg, intravenous immunoglobulin; si-Ctrl, control siRNA; si-HER2, HER2-specific siRNAs; *p < 0.05; **p < 0.01.

To determine the mechanism behind anti-HER2 IVIg effect on OSCC cells, we developed a cell model with siRNA-mediated HER2 knockdown experiments. Three HER2-targeting siRNAs were transfected into CAL27 and SCC25 cells to suppress HER2 expression, in which HER2-specific siRNA-2 showed the maximum of knockdown efficiency (Figures 7A–D). The CCK-8 assay demonstrated that anti-HER2 IVIg could significantly inhibit the proliferation of CAL27 cells (*p* = 4.34 × 10^−5^) and SCC25 cells (*p* = 0.006) treated with control siRNAs compared with regular IVIg and 200 μg/ml trastuzumab. However, this inhibitory effect was vanished in the HER2-specific siRNA-2 transfected cells (Figures 7E,F). The apoptosis assay further confirmed that anti-HER2 IVIg could significantly promote the apoptosis of CAL27 cells (*p* = 0.021) and SCC25 cells (*p* = 0.020) treated with control siRNAs, as compared with regular IVIg and 200 μg/ml trastuzumab, although the effect of promoting apoptosis faded away in the siHER2-treated cells (Figure 7G). The above data proved that HER2 was the target of anti-HER2 IVIg exerting anti-tumor activity.

## Discussion

Natural autoantibodies, including IgM, IgG, and IgA, were discovered nearly half a century ago. Of these three isotypes of natural antibodies, IgG is the most common type and abundant in human plasma (Schwartz-Albiez et al., 2009; Wang et al., 2017). It has been reported that natural autoantibodies are likely to serve as a prominent anti-tumorigenic component in the body to exert immune surveillance against transformed cells (Avrameas et al., 2007; Panda and Ding, 2015; Liu et al., 2021). Plasma levels of natural autoantibodies vary from people to people, and different stages of cancer provide the clues to the insight into risk of malignant tumors and prognostic assessment (Zhao et al., 2018). HER2 belongs to the epidermal growth factor family and plays a key role in cell growth due to high expression in several solid tumors including oral cancer (Connell and Doherty, 2017; Wang and Xu, 2019). HER2 expression has been found to be enhanced in OSCC tissues compared with their adjacent tissues, although the association between anti-HER2 natural autoantibody and OSCC has yet to be established.

In this study, we found that plasma anti-HER2 IgG levels were significantly different among control subjects, individuals with oral benign tumor and patients with OSCC. Individuals with benign tumor or early stage of OSCC had lower levels of plasma natural anti-HER2 antibodies than controls subjects, suggesting that natural anti-HER2 antibodies play a crucial role in preventing tumorigenesis by inhibiting the growth of both OSCC and benign tumors. Plasma anti-HER2 IgG levels were gradually increased with OSCC progression but were not significantly different between two subgroups of OSCC patients in different stages although histological observations also confirmed high expression of HER2 in patients with middle & advanced OSCC. It is possible that with the progression of OSCC, increased HER2 production promotes the secretion of anti-HER2 IgG to prevent further growth of tumors. Zhao et al. suggested that high anticancer autoantibody levels were associated with longer survival in patients with non-small cell lung cancer (Zhao et al., 2018). Accordingly, individuals with a decrease in anticancer autoantibody levels may be at a high risk of developing cancer due to lack of immunological surveillance to monitor transformed cells in the body.

Taken together, natural anti-HER2 IgG levels are likely to impact the tumorigenesis of OSCC based on the present findings that anti-HER2 IgG-abundant plasma could significantly inhibit the proliferation and invasion of OSCC cells, and induce the apoptosis of these cancer cells. The key pharmaco-dynamic mechanism behind anticancer activity of mAb is involved in inhibition of cell signaling, induction of apoptosis, activation of antibody-dependent cell-mediated cytotoxicity (ADCC) and the CDC pathway as well as targeting a toxic payload to tumor cells (Schwartz-Albiez et al., 2008; Schwartz-Albiez et al., 2009; Meyer et al., 2014). Interestingly, the present study demonstrated that the CDC pathway might play a major role in killing OSCC cells by anti-HER2 IgG-abundant plasma treatment. However, there are some unwanted factors present in human plasma, such as growth factors, IgA, and IgE, which may result in adverse outcomes and allergic reactions. Approximately 98% IVIg is IgG prepared from several thousands of healthy blood donors; human IVIg may be a good agent for anticancer therapy and other treatments such as primary humoral immunodeficiency, inflammation and autoimmune diseases (Corbí et al., 2016; Alonso et al., 2020). Both *in vitro* studies and animal models showed that IVIg could inhibit the growth and spreading of several types of solid cancer, such as melanoma, colon cancer, breast cancer and sarcoma (Fishman et al., 2002; Sapir et al., 2005; Schwartz-Albiez et al., 2009). For this reason, anti-HER2 IVIg made from anti-HER2 IgG-abundant plasma was applied to verify its anticancer effects on OSCC cells in this study. Our data suggested that anti-HER2 IVIg could significantly inhibit OSCC proliferation and promote the apoptosis of OSCC cells, and the expression of some apoptosis-associated factors was also influenced, including downregulation of Bcl-2 and upregulation of Bax, Bak, cytochrome c, casepase-9, casepase-3, and cleaved casepase-3. Although there are several therapies available for clinical treatment of OSCC, the rates of recurrence and metastasis are still very high, which is the most reasons for the poor prognosis of OSCC patients. Based on this study, anti-HER2 IVIg is much stronger to kill OSCC cells than tastuzumab that is currently recommended as a first-line treatment of patients with metastatic HER2-positive breast cancer (Rochette et al., 2015; Kristeleit et al., 2016), as our data showed that trastuzumab had almost no inhibitory effect on OSCC cells and that inhibitory effect of anti-HER2 IVIg on breast cancer cells was more powerful than trastuzumab. Since trastuzumab is produced in humanized mice, there are still 30% homologous to mice, leading to serious side effects, in which cardiotoxicity and drug resistances are the most common issues (Nemeth et al., 2017). It has been reported that trastuzumab can cause symptomatic congestive heart failure (CHF) and develop severe drug resistance within 1 year of medication (Rochette et al., 2015). However, anti-HER2 IVIg is a natural autoantibody originated from healthy individuals and is unlikely to produce serious side effects and treatment resistance. As mentioned above, IVIg is a mixture of allogeneic IgG antibodies and the inhibitory effect of anti-HER2 IVIg on OSCC cells has been confirmed by work on HER2-knockdown OSCC cells. To our knowledge, this is the first report on the role of plasma anti-HER2 IgG and anti-HER2 IVIg in inhibiting OSCC growth and invasion.

While plasma anti-HER2 IgG levels are likely to be associated with OSCC progression and anti-HER2 IVIg may have a potential for OSCC therapy because of its anti-OSCC properties, there are a few limitations of this study. First, the sample size for clinical study was too small to draw a firm conclusion; replication of this initial finding is needed with large sample collection. Second, clinical information was incomplete and the overall survival of OSCC patients with different plasma anti-HER2 IgG levels need investigating. Third, the cell model developed in this study may not be strong enough to reflect an *in vivo* change, so that the effect of anti-HER2 IVIg on OSCC should be replicated in patient-derived tumor xenograft (PDX) mouse model.

## Conclusion

In summary, the present results indicate that plasma anti-HER2 IgG and anti-HER2 IVIg preparations have significantly inhibitory effects on the proliferation and invasion of OSCC cells and the CDC pathway is likely to be involved in anticancer mechanism. Anti-HER2 IVIg may be a promising agent for the treatment of OSCC.

## Data Availability

The raw data supporting the conclusion of this article will be made available by the authors, without undue reservation.
