# Supplementary material for: Circulating Natural Autoantibodies to HER2-Derived Peptides Performed Antitumor Effects on Oral Squamous Cell Carcinoma
Source: Front Pharmacol. 2021 Nov 5;12:693989. doi: 10.3389/fphar.2021.693989 (PMC8602057; doi:10.3389/fphar.2021.693989)
Supplement: Supplementary file 1 [file DataSheet1.docx]

Supplementary Material

# Supplementary Materials and Methods

**Cell culture and cell viability assay**

Two cell lines derived from breast cancer, SK-BR-3 and BT-474 (National Infrastructure of Cell Line Resource, China), were both cultured in Iscove's Modified Dulbecco’s Medium (IMDM, Gibco) containing 10% FBS. In addition, BT-474 cells were cultured with 0.01 mg/ml insulin (M&C Gene Technology, Beijing, China). Both cell lines were cultured in humidified atmosphere with 5% CO_2_ at 37 ^o^C.

Cell proliferation was analyzed using a Cell Counting Kit-8 (CCK-8) assay. Briefly, breast cancer cells were seeded in a 96-well plate, and incubated for 48 h with the medium that contains 20% anti-HER2 IgG-abundant or -deficient plasma or 20% anti-HER2 IgG-deficient plasma with trastuzumab and IVIg. Cell viability was used to present data and calculated as follows:

$$Cell Viability=\frac{{OD}_{abundant}-{OD}_{blank}}{{OD}_{deficient}-{OD}_{blank}}$$

# Supplementary Figures and Tables

## Supplementary Tables

**Supplementary Table 1. Primary antibodies for Western blot assay**

| Primary antibody | Item No. | Manufacturer | Country |
| --- | --- | --- | --- |
| E-Cadherin rabbit mAb | #3195 | CST | USA |
| Vimentin rabbit mAb | #ab92547 | Abcam | USA |
| Snail rabbit pAb | #ab180714 | Abcam | USA |
| Bcl-2 rabbit mAb | #2870 | CST | USA |
| Bax rabbit mAb | A19684 | ABclonal | China |
| Bak rabbit mAb | A0498 | ABclonal | China |
| Cytochrome c Rabbit mAb | #11940 | CST | USA |
| Caspase-9 rabbit mAb | A18676 | ABclonal | China |
| Cleaved caspase-3 rabbit mAb | #9664 | CST | USA |
| Caspase-3 rabbit mAb | #ab32351 | Abcam | USA |
| HER2 rabbit pAb | A2071 | ABclonal | China |
| β-actin rabbit mAb | AC026 | ABclonal | China |
| Histone H3 rabbit mAb | #10809 | Santa Cruz Biotechnology | Canada |

**Supplementary Table 2. Primer sequences used for analysis of HER2-specific siRNAs by quantitative real-time PCR**

| siRNA | Sequences (5’-3’) |
| --- | --- |
| si-HER2-1 | Sense: GCAGUUACCAGUGCCAAUATT Antisense: UAUUGGCACUGGUAACUGCTT |
| si-HER2-2 | Sense: GGUGUAUGCAGAUUGCCAATT Antisense: UUGGCAAUCUGCAUACACCTT |
| si-HER2-3 | Sense: GCUCUUUGAGGACAACUAUTT Antisense: AUAGUUGUCCUCAAAGAGCTT |
| Negative control siRNA | Sense: UUCUCCGAACGUGUCACGUTT Antisense: ACGUGACACGUUCGGAGAATT |

**Supplementary Table 3. Kolmogorov–Smirnov test for a normal distribution of plasma IgG levels**

| Group | Skewness | Kurtosis | *p* |
| --- | --- | --- | --- |
| Control | 1.235 | 3.919 | 0.007 |
| Benign | 1.275 | 3.096 | 0.025 |
| OSCC | 1.058 | 1.301 | 0.000 |


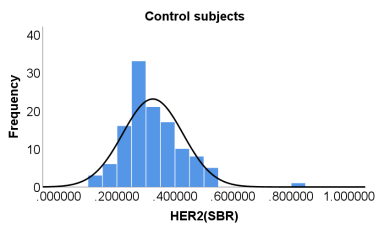

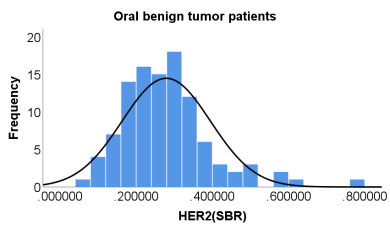

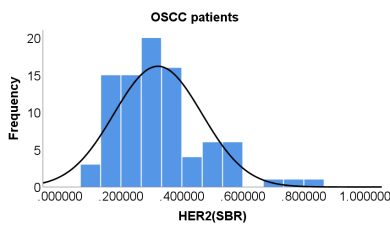


**Supplementary Table 4. The inner-assay deviation estimated by analysis of QC samples (SBR)**

| IgG | Mean±SD | N^a^ | CV (%)^b^ |
| --- | --- | --- | --- |
| Anti-HER2 | 0.67±0.02 | 15 | 2.75 |

^a^ The number of plates tested

^b^ Coefficient of variation

**Supplementary Table 5. Binary logistic regression analysis of plasma anti-HER2 IgG lelves between control subjects, benign patients and OSCC patients**

| Group | Control-OSCC | | |  | Benign-OSCC | | |
| --- | --- | --- | --- | --- | --- | --- | --- |
|  | B | Wald | *p*^a^ |  | B | Wald | *p*^a^ |
| HER2(SBR) | -0.453 | 0.103 | 0.748 |  | 1.899 | 2.267 | 0.132 |
| Age | 0.169 | 37.882 | 0.000 |  | 0.067 | 27.176 | 0.000 |
| Gender | -0.439 | 1.619 | 0.203 |  | 0.251 | 0.603 | 0.437 |

^a^*p*<0.05 was considered to be statistically significant.

## Supplementary Figures


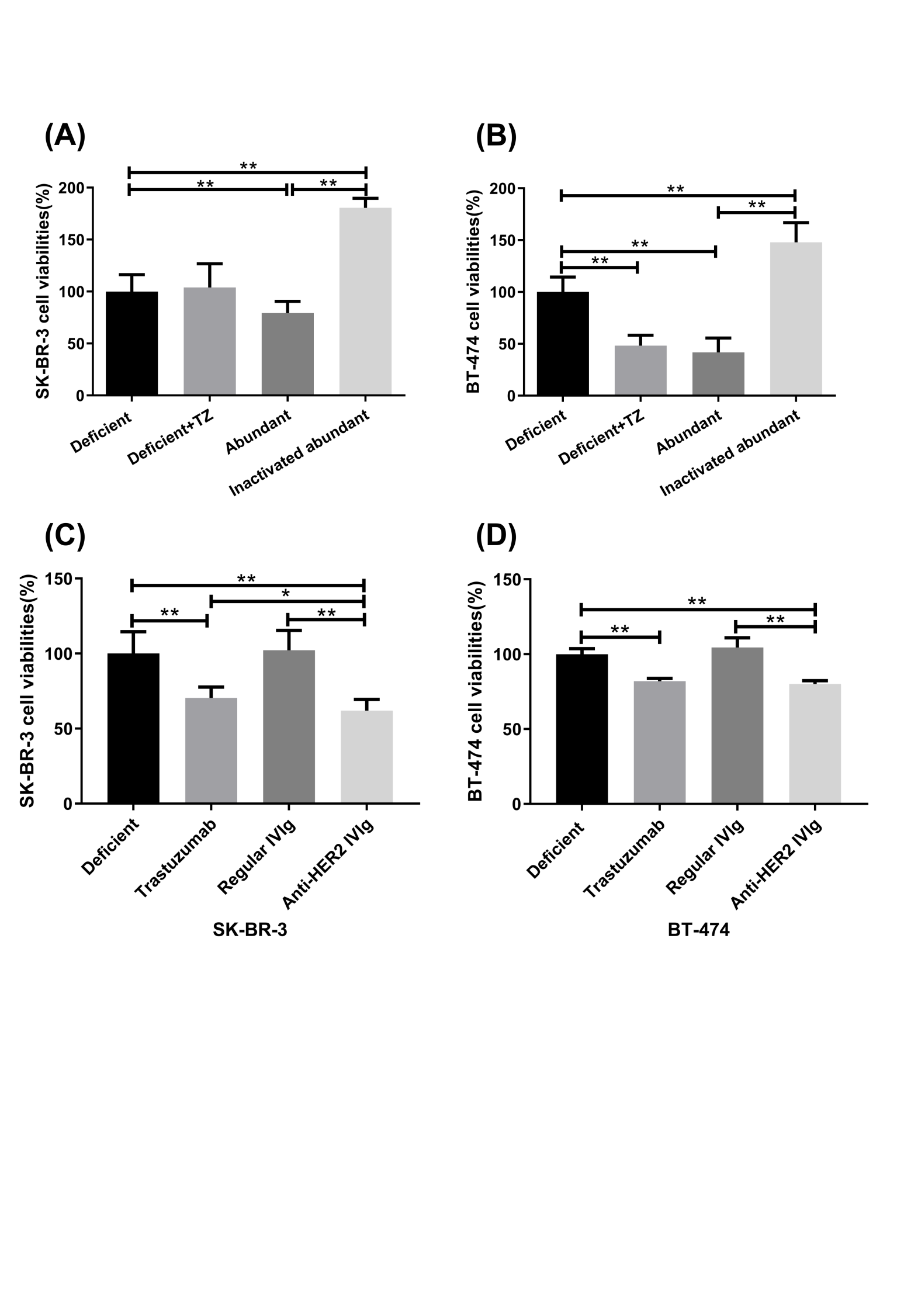


**Supplementary Figure 1.** Plasma anti-HER2 IgG and anti-HER2 IVIg inhibited the proliferation of breast cancer cells. **(A, B)** SK-BR-3 and BT-474 cells were treated for 48 h with anti-HER2 IgG-deficient plasma only, anti-HER2 IgG-deficient plasma containing 200μg/ml of trastuzumab, anti-HER2 IgG-abundant plasma and inactivated anti-HER2 IgG-abundant plasma, respectively. **(C, D)** Viability of SK-BR-3 and BT-474 cells treated with anti-HER2 IgG-deficient plasma only, 200μg/ml of trastuzumab, 2.5mg/ml regular IVIg and 2.5mg/ml anti-HER2 IVIg, respectively. The data were expressed as mean ± SD in cell viability (%). TZ: trastuzumab; IVIg: intravenous immunoglobulin; **p*<0.05; ***p*<0.01.


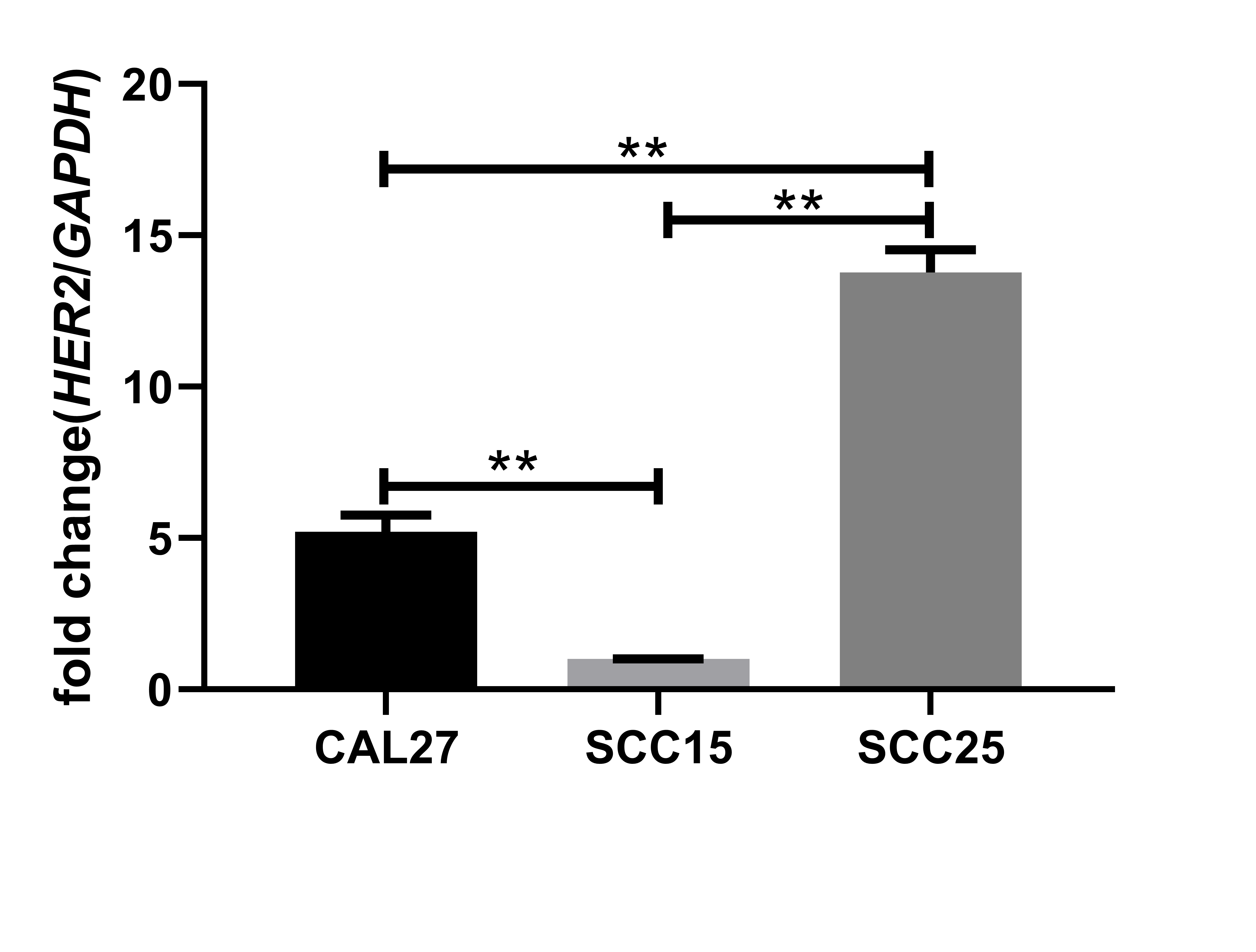


**Supplementary Figure 2.** Expression of HER2 mRNA in OSCC cells. The data were expressed as mean ± SD in fold change. **p*<0.05; ***p*<0.01.
